# Structural Diversity of Di-Metalized Arginine Evidenced by Infrared Multiple Photon Dissociation (IRMPD) Spectroscopy in the Gas Phase

**DOI:** 10.3390/molecules26216546

**Published:** 2021-10-29

**Authors:** Ruxia Feng, Yicheng Xu, Xianglei Kong

**Affiliations:** 1 State Key Laboratory and Institute of Elemento-Organic Chemistry, College of Chemistry, Nankai University, Tianjin 300071, China; fengruxia@126.com (R.F.); 2120190792@mail.nankai.edu.cn (Y.X.); 2 Collaborative Innovation Center of Chemical Science and Engineering, Nankai University, Tianjin 300071, China

**Keywords:** IRMPD spectroscopy, metallization, amino acid, structural diversity, mass spectrometry

## Abstract

Although metal cations are prevalent in biological media, the species of multi-metal cationized biomolecules have received little attention so far. Studying these complexes in isolated state is important, since it provides intrinsic information about the interaction among them on the molecular level. Our investigation here demonstrates the unexpected structural diversity of such species generated by a matrix-assisted laser desorption ionization (MALDI) source in the gas phase. The photodissociation spectroscopic and theoretical study reflects that the co-existing isomers of [Arg+Rb+K−H]^+^ can have energies ≥95 kJ/mol higher than that of the most stable one. While the result can be rationalized by the great isomerization energy barrier due to the coordination, it strongly reminds us to pay more attention to their structural diversities for multi-metalized fundamental biological molecules, especially for the ones with the ubiquitous alkali metal ions.

## 1. Introduction

Metal ions are essential for living processes by regulating structures and functions of various biological molecules, including amino acids, peptides, proteins, and nucleic acids [1,2,3,4,5]. They also play an important role in synthetic chemistry relative to those biomolecules [6]. Experimental and theoretical studies on the metal-biomolecule complexes in isolated state are very important to the issue since they can provide unique and intrinsic information about the interaction between the metal ion and the biomolecule on the molecular level [7,8,9,10]. Among them, infrared multiple photon dissociation (IRMPD) spectroscopy, combined with mass spectrometry, provides valuable information about their structures and interactions and have been widely applied in the field [11,12,13,14,15,16,17,18,19,20,21,22,23,24]. For example, the interaction of metal ions with amino acids and peptides have been extensively studied and the internal rules relative to their structural preference have been gradually revealed in the past 20 years [17,18,19,20,21,22,23,24,25]. However, most of these studies only focus on the singly metalized complexes, and we still know little about such complexes with multiple metal ions. Considering that metal ions including Na^+^ and K^+^ have concentration-dependent effects on chemical properties of amino acids in cell [26], multiply metalized complexes, just as the singly metalized counterparts, need further investigation. It is also known that the bimetallic complexes can provide different chemical properties and reactivity patterns from their monometallic counterpart in organic chemistry [27]. Thus, a detailed structural study on such di-metalized complexes is a good test. Herein we report the first gas phase IRMPD spectrum of the heteronuclear bimetallic complex ion of Arginine (Arg). The complex ion of [Arg+Rb+K−H]^+^ was chosen as the example for this study, since the ion can be generated herein with a better reproducibility than other observed bimetallic complex ions, such as [Arg+2Na−H]^+^ and [Arg+2K−H]^+^. This research aims at a better understanding for the interaction and structure of the species, based on the experimental IRMPD method and theoretical calculations. The results can further introduce the study of micro solvation of such bimetallic ions on the single molecule level, which bridges the structure and property study for such molecules in the gas and condensed phases.

## 2. Results

**Figure 1:**
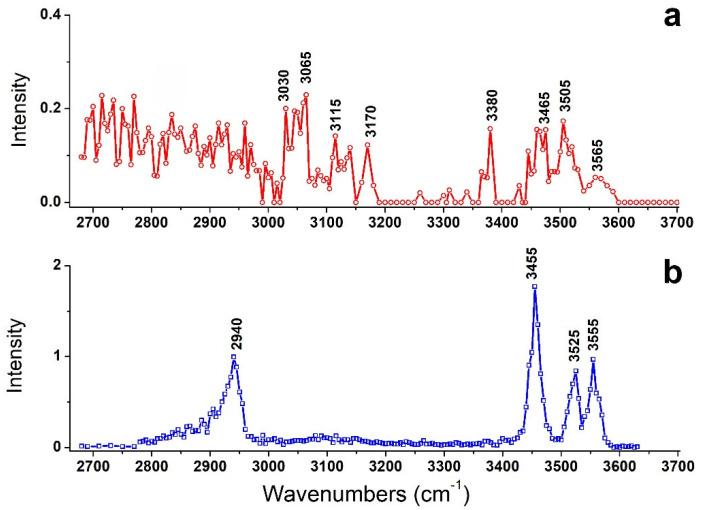
IRMPD spectra of (**a**) [Arg+Rb+K−H]^+^ and (**b**) [Arg+Rb]^+^. The latter is taken from ref [16].

Under suitable IR wavelengths, the absorption of IR photons can induce the fragmentation of the precursor ions. As shown in Figure S1, two fragment ions were observed for the target ions of [Arg+Rb+K−H]^+^. One is the fragment ion due to the loss of NH_2_ group, and the other is Rb^+^. By tuning output wave-numbers of the optical parametric oscillator (OPO) laser, IRMPD mass spectra of the precursor ion in the region were recorded in a step of 5 cm^−1^, thus the action spectrum of the target ion was obtained (Figure 1a).

For comparison, the previously observed IRMPD spectrum of [Arg+Rb]^+^ is also shown here as Figure 1b. Interestingly, their IRMPD spectra are quite different from each other. The spectrum of [Arg+Rb]^+^ is clearly characterized by sharp peaks at 2940, 3455, 3525 and 3555 cm^−1^, while that of [Arg+Rb+K−H]^+^ shows relatively weak peaks at 3030, 3065, 3115, 3170, 3380, 3465, 3505 and 3565 cm^−1^, accompanied with broad absorptions in the region of 2680–3000 cm^−1^.

**Figure 2:**
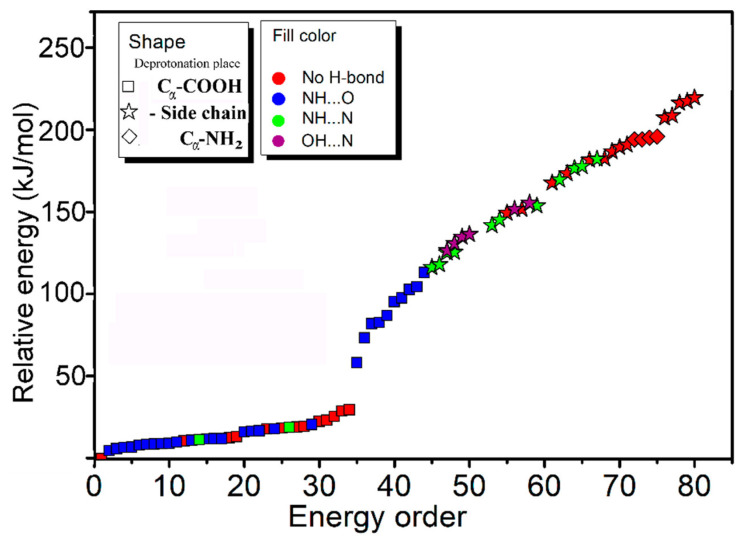
A statistical view on the 80 isomers of [Arg+Rb+K−H]^+^ corresponding to their energy orders based on the method of B3LYP/6-311++G(d,p)~LAN and their structural characteristics.

**Figure 3:**
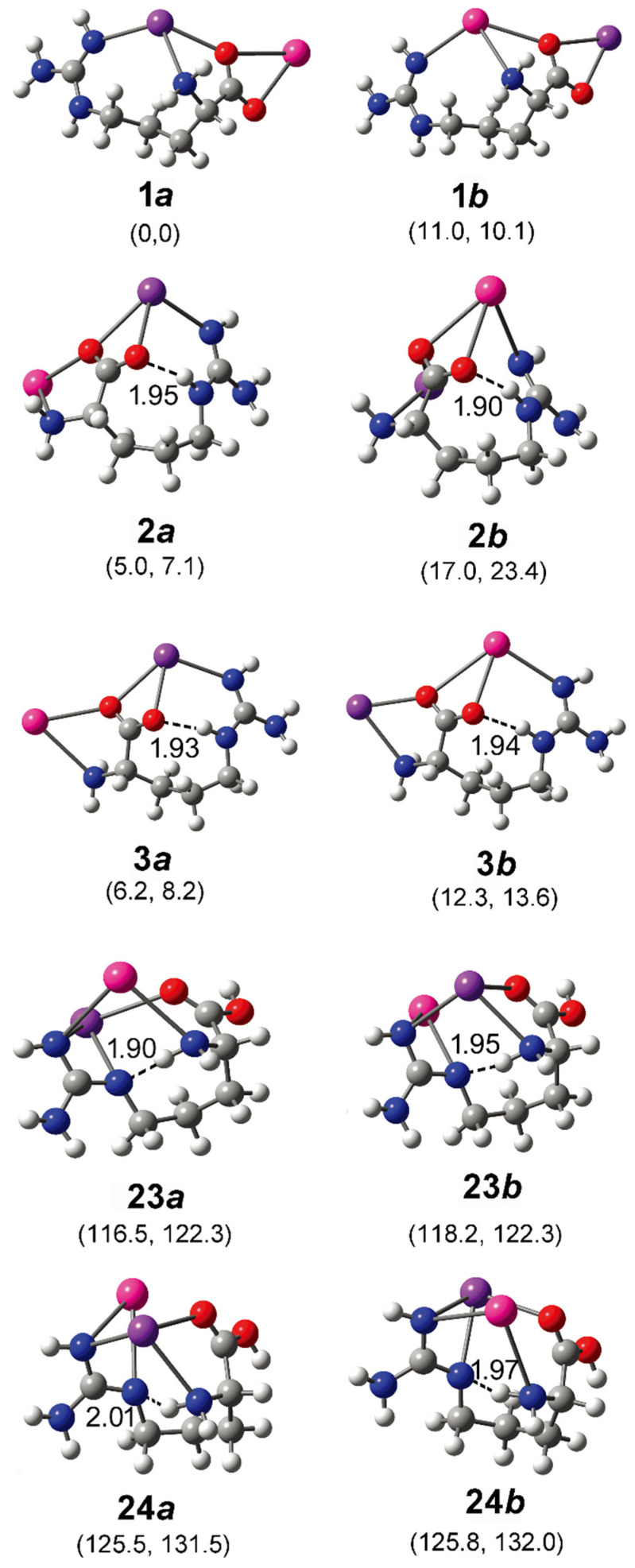
Some optimized isomers of [Arg+Rb+K−H]^+^ at the level of B3LYP/6-311++G(d,p)~LAN. The most stable isomer is identified as **1*a***. The relative energies at 0 K and Gibbs energies at 298 K relative to those of **1*a*** are shown in the parentheses below each subfigure (in kJ/mol). The distance of each intramolecular H-bond (in Å) is shown too. The pair of isomer ***a*** and ***b*** are obtained by exchanging the positions of Rb and K atoms and re-optimization. The atoms of K, Rb, O, N, C, and H are colored by purple, pink, red, blue, gray, and light gray, in turn.

In order to better understand the results, systematic theoretical calculations relative to the most stable isomers of [Arg+Rb+K−H]^+^ were investigated by a self-developed procedure based on the density functional theory (DFT) method of B3LYP. The 80 isomers with relative energies no more than 230 kJ/mol to the most stable isomer were identified at the level of B3LYP/ 6-311++G(d,p)~LAN (Table S1). These structures are summarized in Figure 2 and Figure S2. Figure 2 shows a statistical view on the calculated isomers corresponding to their energy orders and structural characteristics. As shown there, the top 45 isomers are all characterized by a deprotonation place at C_α_-COOH, and most of them own one intramolecular NH…O H-bond. The three most stable isomers of **1*a***, **2*a*** and **3*a*** are listed and shown in Table 1 and Figure 3, respectively. Among them, the most stable isomer **1*a*** has a structure free of intramolecular H-bond. The distance between the two metal atoms extends to 5.35 Å to reduce the Coulomb repulsion. Interestingly, each structure has a twin isomer, which is formed by exchanging the positions of Rb and K atoms. For convenience, the twin isomers are respectively named as ***a*** and ***b*** here, in which the one with lower energy is identified as ***a***. As shown in Figure 3 and Table 1, the twins of **1*a*** and **1*b*** have very similar structures except the positions of two metal atoms. However, the isomer **1*b*** has an energy 5.0 kJ/mol higher than that of **1*a***.

For the isomer **2*a*** with the second lowest energy, the distance of Rb and K atoms (5.27 Å) is very close to that of **1*a***. But the structure is characterized a NH…O H-bond with a length of 1.95 Å and an angle of 158.7°. A different aspect is that its twin isomer **2*b*** is somewhat different from **2*a*** after the optimization. The structure is more compactly folded and thus the distance of the metal atoms reduces to 4.51 Å, accompanied by a small change of the intramolecular H-bond. The isomers of **3*a*** and **3*b*** are similar to that of **2*a***, except that the metal–metal distance increases to 5.4/5.5 Å. Other low-energy isomers also generally have similar structures to that of **3*a*** (Figure S2).

These isomers were also optimized with B3LYP based on a more size-consistent basis set of def2-TZVP. Both structures and relative energies are very similar to those calculated in B3LYP/6-311++G(d,p)~LAN level, as shown in Table 1. To further check the results, the method of MP2 was also applied for the optimization and frequency analysis of these isomers. The optimized isomers have structures very close to those shown in Figure 3. As shown in Table 1, the results obtained based on the two methods are generally consistent with each other. Based on the MP2 method, the isomer **1*a*** is still the most stable one, with the energy 16.3 kJ/mol lower than that of **1*b***. The distances between the two metal atoms in **1*a*** and **1*b*** are 5.34 and 5.25 Å, respectively. For isomer **2*a***, its energy is 2.5 kJ/mol higher than that of **1*a***. The M06-2X method also reports a similar NH…O H-bond with a length of 1.93 Å and an angle of 154.1°, and a closer Rb-K distance of 5.18 Å than that one reported by the method of B3LYP in structure **2*a***. However, the relative energy of **2*b*** is found to be only 2.6 kJ/mol, much lower than the value of 17.2 kJ/mol obtained with the B3LYP method. A shorter H-bond length is reported to be 1.82 Å with an angle of 163.8°. For isomers **3*a*** and **3*b***, very similar results have been obtained for both methods.

**Figure 4:**
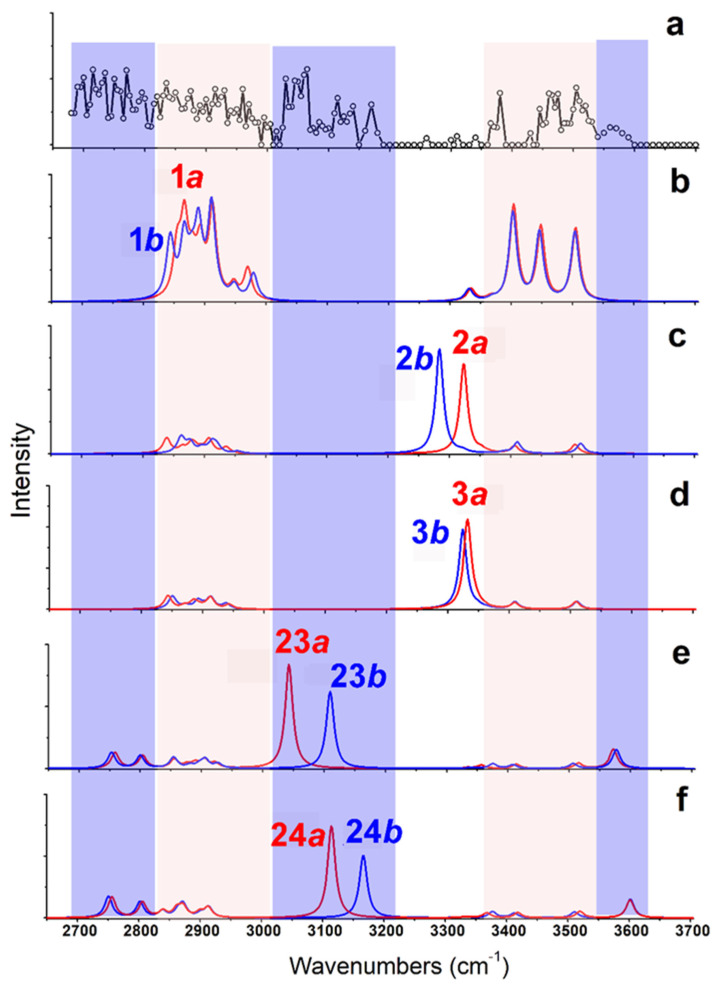
(**a**) Experimental IRMPD spectrum of [Arg+Rb+K−H]^+^ and (**b**–**f**) the calculated vibrational spectra of different isomers obtained at the level of B3LYP/6-311++G(d,p)~LAN. The structures of these isomers are shown in Figure 3.

As shown in Figure 4, the calculated IR spectra of **1*a*** and **1*b*** (based on B3LYP/6-311++G(d,p)~LAN) are very similar to each other. The predicted band in the region of 3350–3550 cm^−1^ (from NH stretches) corresponds to experimental results well (Restricted by the S/N ratios of the experimental spectrum, a quantitative description of the frequencies and relative intensities of IR vibrations in such complexes was not performed here [28,29].) For the experimentally observed broad band 2680–3000 cm^−1^, the predicted spectra only show strong and weak absorptions in two regions of 2820–2930 cm^−1^ and 2930–3000 cm^−1^ (both from CH stretches). On the other hand, the calculated IR spectra of **2*a***/**2*b*** and **3*a***/**3*b*** are similar, but poorly agree with the experimental results in the region 3250–3350 cm^−1^. All those spectra show strong absorption at ~3300 cm^−1^ coming from the vibrations of hydrogen bound NH bond, which are obviously absent in the experimental spectrum. The calculated IR spectra based on the methods of B3LYP/def2-TZVP and MP2/6-311++G(d,p)~SDD show similar results. The results obtained by the MP2 method are shown in Figure S3.

Obviously, some bands in the experimental spectrum, such as the absorption at 3565 cm^−1^ and the band in the region 3020–3200 cm^−1^, cannot be explained by these low-energy isomers. Even considering all the top 30 isomers (Figure S4), their predicted spectra are all absent in these characteristics. Thus, the experimentally generated ions should include some isomers with different structures. Considering that the observed peak at 3565 cm^−1^ is from the vibrations of free carboxylic OH vibrations [30,31,32], there must exist isomers with the group of -COOH. This is very unexpected, since all previous experimental and calculational proofs clearly indicated that Arg is very likely to form zwitterionic structures when interacted with metal ions, or other molecules in the gas phase [33,34,35,36]. A further check on the calculated isomers of [Arg+Rb+K−H]^+^ (Figure 2 and Table S1) also shows that the isomers including the -COOH group are typically 125–250 kJ/mol higher in energy than the most stable isomer of **1*a***.

Based on the method of B3LYP/6-311++G(d,p)~LAN, among these isomers including a group of -COOH, the most stable one is **23*a***, which has an energy 116.5 kJ/mol higher than **1*a*** (Figure 3). The structure is characterized by a free -COOH, a NH…N H-bond (1.90 Å) and a relatively short distance between the two metals (4.06 Å). Its twin isomer **23*b*** and other relative isomers (**24*a***, **24*b*** and others) have similar structures (Figure 3 and Figure S2). Both predicted spectra of **23*a*** and **23*b*** show their free carboxylic OH vibrations at ~3575 cm^−1^ and weak absorptions at the nearby of 3360, 3410, and 3515 cm^−1^ due to the NH stretch, the symmetric and antisymmetric stretches of the NH_2_ group. Remarkably, the spectra of **23*a*** and **23*b*** show strong absorptions at 3042 and 3110 cm^−1^ that are assigned as the intramolecular H-bonded NH stretches, respectively. For the isomer **24*a*** and **24*b***, similar spectroscopic characteristics, including the free carboxylic OH at ~3600 cm^−1^ and the intramolecular H-bonded NH vibrations at 3115 and 3165 cm^−1^ are predicted. Obviously, the combination of these peaks agrees with the experimental spectrum in the regions of 3010–3200 cm^−1^ and 3550–3600 cm^−1^, which cannot be explained by the isomer of **1*a*** and its relatives.

CH stretches are normally considered of little diagnostic value in structural analysis. However, an inspection of the experimental spectrum in the low wavenumber region is very helpful here. Although the observed absorptions in the region of 2820–3010 cm^−1^ can be assigned to be CH stretches of **1*a***/**1*b***, the absorptions below 2820 cm^−1^ do not. Considering the small size of the ion, the intramolecular H-bonded groups of NH or OH are restricted very much, which can be hardly red-shifted to this region. Unexpectedly, the predicted CH bands of **23*a***/**23*b*** and **24*a***/**24*b*** have significantly expanded to ~2730 cm^−1^, although the observed absorption below 2730 cm^−1^ is still puzzling. Overall, the experimental and calculational results support the co-existence of both type isomers of **1** and **23**/**24**.

The suggestions are also supported by the calculation based on the MP2 method. As shown in Table 1, the structures of **23**/**24** obtained with the two methods are very similar. Their relative energies based on the MP2 method are typically 21–25 kJ/mol lower than those based on the B3LYP/6-311++G(d,p)~LAN method. The predicted vibrational spectra of them are also very similar to those shown in Figure 4 (Figure S3).

## 3. Discussion

Even if the co-existence of multiply isomeric ions in the gas phase has been previously proven [37,38,39,40,41,42], the large energy difference between the two co-existing isomers reported here (≥95 kJ/mol based on the MP2 method) is still very surprising for such a small-sized ion. Solvation effect was also considered in water by employing the integral equation formalism polarizable continuum model (IEFPCM) [43]. Similar results were obtained, in which the isomers **23*a*** and **23*b*** both had energies 111 kJ/mol higher than **1*a***. The ratios of these high-energy isomers cannot be directly explained by their Boltzmann distribution. On the other hand, considering the amino acid has a zwitterionic structure in the solid or solution phase, it cannot be rationalized by the memory effect of the structure before the ionization. A conjecture for the results is that the rapid process of metal coordination from the initially generated species hinders the subsequent processes of isomerization (unlike organic matrices, graphene can generate a laser plume with metal cations in high density [25]). The consecutive metallization happens so quickly that the thermodynamic driven processes of isomerization do not have enough time to fully occur. After the bimetal coordination, the complex ions become much less flexible, since the rotation of the C-C bond is restricted by the coordination of metals, which also results in high energy barriers for isomerization. However, further computational investigation is still needed for a better understand of its potential energy surface and isomerization barriers. On the other hand, how the replacement of Rb^+^ with Na^+^ or K^+^ can affect their structures, energies, and isomerization processes for such complexes is a very attractive question. Previous results about singly alkali metal cationized amino acids show that the arginine changes from its nonzwitterionic to zwitterionic form between lithium and sodium, indicating the metal ion size can highly affect their structures [19]. At the same time, because the charge densities of Na^+^ and K^+^ can lead to larger repulsive forces than Rb^+^, Coulomb interaction may give priority to structures with longer distances of two metal atoms (such as **1*a***) in such di-metalized species, making other structures (such as **23*a***) less stable (which may be one of the reasons why the signals of such species were weak in our experiments). However, considering the ubiquitous existence of sodium or potassium and their concentration-dependent effects on amino acids in cells [26], these di-metalized species are worthy of further study.

## 4. Materials and Methods

Sample of graphene was purchased from Timesnano Company (Beijing, China). l-arginine (Arg), KCl and RbCl were obtained from Sigma-Aldrich (St. Louis, MO, USA). The solution of Arg was prepared with a concentration of 2 mM in CH_3_CN/water (1:1, *v*/*v*), together with 0.2 mM RbCl and 0.2 mM KCl. The sample of graphene was suspended in acetone containing 15% water with a concentration of 1 mg/mL. After a process of sonication for 10 min, 0.5 μL graphene suspension was pipetted and spotted on the metal target in the first step. After it dried, 0.5 μL of Arg with RbCl and KCl was dropped on it in the second step. The target was then dried in the air and sent into the matrix-assisted laser desorption ionization (MALDI) source for experiments.

The experimental setup has been described in our previous paper [16]. Briefly, an IR OPO laser (M Squared Lasers Ltd., Glasgow, UK) was combined with a 7.0 T Fourier-transform ion cyclotron resonance mass spectrometer (FT-ICR-MS) (Varian, Inc., Lake Forest, CA, USA). The ions are generated by a MALDI source equipped with a 355 nm Nd:YAG laser (Orion, New Wave, Fremont, CA, USA) at a typical pulse energy of 2.4 mJ. The OPO has a typical line width of 7 cm^−1^ and can be tunable in the range from 2680–4000 cm^−1^ with an average power at 160 mW. In the experiments, ions produced by six consecutive laser pulses were accumulated in the hexapole first. After the accumulation, the hexapole exit lens was gated so that ions could be transferred into the cell of FT-ICR. The target ions were selected by the method of stored waveform inverse Fourier transform (SWIFT) [42] and then trapped in the cell for laser irradiation. The irradiation time of the laser is set as 40 s and controlled with a mechanical shutter (Sigma-Koki, Tokyo, Japan). No focus lens is used in the experiment. During the process of IR irradiation, the cell of FT-ICR kept high vacuum with a pressure less than 4 × 10^−10^ Torr, indicating that the collision-induced dissociation or secondary reaction can be neglected. However, due to the long irradiation time, sequential dissociation of primary fragment ion is also possible in the process. The IRMPD spectra were recorded with a step size of 5 cm^−1^. The spectral intensity at each wavelength is calculated with the same method described previously [16].

Systematic theoretical calculations of [Arg+Rb+K−H]^+^ were investigated by a self-developed procedure shown in Scheme S1. Briefly, based on the initial structures of [Arg+K]^+^ previously reported [32], the isomers with all possible seven deprotonation sites were considered and then the second metal was added. To make the calculation on AM1 method performable, we used two K atoms at the first step. After the selection of the top energy isomers, one K atom were replaced by one Rb atom and reoptimized. At last the 80 (40 pairs of twins) isomers were optimized and selected on the level of B3LYP/6-311++G(d,p)~LAN [32,44,45,46]. Frequencies of all these structures were also calculated on the same level. Their electronic energies were calculated at 0 K with zero-point energy corrections and Gibbs energies were calculated at 298 K. Methods of B3LYP/def2-TZVP and MP2/6-311++G(d,p)~SDD were further applied for the optimization and frequency analysis of some selected isomers [47,48]. To get their predicted IR spectra, scaling factors of 0.952 and 0.940 were used for the B3LYP and MP2 methods, respectively. All calculations were carried out using the Gaussian 09 program package [49]. The Cartesian coordinates of some structures (**1*a*/*b***~**3*a*/*b***, **23*a*/*b***, **24*a*/*b***) are shown in Table S2.

## 5. Conclusions

In summary, [Arg+Rb+K−H]^+^, as one example of the di-metal cationized molecules that have received little attention so far, was generated in the gas phase with the matrix of graphene and investigated by IRMPD spectroscopy and theoretical calculations. Its IRMPD spectrum in the region of 2680–3700 cm^−1^ was found to be very complicated. For the hetero di-metal cationized species, each stable isomer was found to be accompanied by a twin isomer, characterized by exchanging the positions of the two different metal ions. Spectroscopic and calculational evidences show that the most stable isomer of [Arg+Rb+K−H]^+^, **1*a***, co-exists with some unexpected isomers, including the -COOH group, which have energies 95 kJ/mol higher than that of the former. The phenomenon is unverified, suggesting it could be rationalized by rapid processes of metallization in the ion source, resulting in blocking subsequent isomerization, since the coordination greatly restricts the rotation of relative sigma bonds. As alkali metal ions are prevalent in organic solvent or biological media, the present results provide a new perspective into the generation, stabilization, and existence of high-energy isomers of metal cationized amino acids and their relatives, which might affect their functions and reactivities. However, to further understand how structural diversity can be affected by solvents, and how gas phase structures can be related to ones in solution or gas-liquid interfaces, studies on the micro solvation of such bimetallic ions should be investigated systemically in further steps.

## Figures and Tables

**Table 1:** Relative energies and structural parameters of some isomers obtained with different methods.

Isomers	Methods	ΔE (kJ/mol)	ΔG (kJ/mol)	H Bond (Å)	*d_Rb-k_* (Å)
**1*a***/**1*b***	A	0/11.0	0/10.1	None	5.35/5.29
	B	0/10.0	0/9.2	None	5.29/5.24
	C	0/16.3	0/16.3	None	5.34/5.25
**2*a***/**2*b***	A	5.0/17.0	7.1/23.4	NH…O (1.95/1.90)	5.27/4.51
	B	5.0/15.5	6.9/22.2	NH…O (1.97/1.89)	5.23/4.46
	C	2.5/2.9	5.0/10.9	NH…O (1.93/1.82)	5.18/4.55
**3*a***/**3*b***	A	6.2/12.3	8.2/13.6	NH…O (1.93/1.94)	5.42/5.55
	B	7.0/11.6	8.6/12.9	NH…O (1.94/1.93)	5.37/5.45
	C	5.9/15.9	8.9/18.4	NH…O (1.96/1.94)	5.36/5.46
**23*a***/**23*b***	A	116.5/118.2	122.3/122.3	NH…N (1.90/1.95)	4.06/4.05
	B	117.9/116.9	123.5/121.6	NH…N (1.92/1.97)	4.02/4.01
	C	95.0/95.0	101.7/100.8	NH…N (1.84/1.92)	4.11/4.06
**24*a***/**24*b***	A	125.5/125.8	131.5/132.0	NH…N (2.01/1.97)	4.16/4.13
	B	124.2/125.5	130.1/131.0	NH…N (2.02/2.00)	4.12/4.11
	C	99.2/99.2	105.0/107.5	NH…N (2.06/2.03)	4.31/4.36

A: B3LYP/6-311++G(d,p)~LAN, B: B3LYP/def2-TZVP and C: MP2/6-311++G(d,p)~SDD. The distances between the two metal atoms in all isomers are indicated as ***d_Rb-k_***. The ΔG was calculated at temperature of 298 K.

## Data Availability

Data is contained within the article.

## Supplementary Materials

The following are available online. Scheme S1: calculation procedure, Table S1: relative energies of the 80 isomers, Table S2: Cartesian coordinates of some structures (**1*a*/*b***~**3*a*/*b***, **23*a*/*b***, **24*a*/*b***), Figure S1: mass spectra, Figure S2: structures of the 80 isomers, Figure S3: IR spectra of some isomers based on the MP2 method, Figure S4: the predicted vibrational spectra of the top isomers of **4*a*/*b***~**16*a*/*b***.

## Sample Availability

Samples of the compounds are available from the authors.
