# Supplementary material for: Structural Diversity of Di-Metalized Arginine Evidenced by Infrared Multiple Photon Dissociation (IRMPD) Spectroscopy in the Gas Phase
Source: Molecules. 2021 Oct 29;26(21):6546. doi: 10.3390/molecules26216546 (PMC8587954; doi:10.3390/molecules26216546)
Supplement: Supplementary file 1 [file molecules-26-06546-s001.zip › molecules-1412431-supplementary.pdf]

molecules

Supplementary Material

Structural Diversity of Di-Metalized Arginine Evidenced by Infrared Multiple Photon Dissociation (IRMPD) Spectroscopy in the Gas Phase

Ruxia Feng [1], Yicheng Xu [1] and Xianglei Kong [1,2,*]

[1] State Key Laboratory and Institute of Elemento-Organic Chemistry, College of Chemistry, Nankai University, Tianjin 300071, China fengruxia@126.com (R.F.); 2120190792@mail.nankai.edu.cn (Y.X.)
[2] Collaborative Innovation Center of Chemical Science and Engineering, Nankai University, Tianjin 300071, China
* Correspondence: kongxianglei@nankai.edu.cn; Tel.: +86-22-23-509-564

Content

Scheme S1 S2

Table S1 S2

Figure S1 S3

Figure S2 S4,S5

Figure S3 S6

Figure S4 S7,S8

Table S2 S9

Reference S14

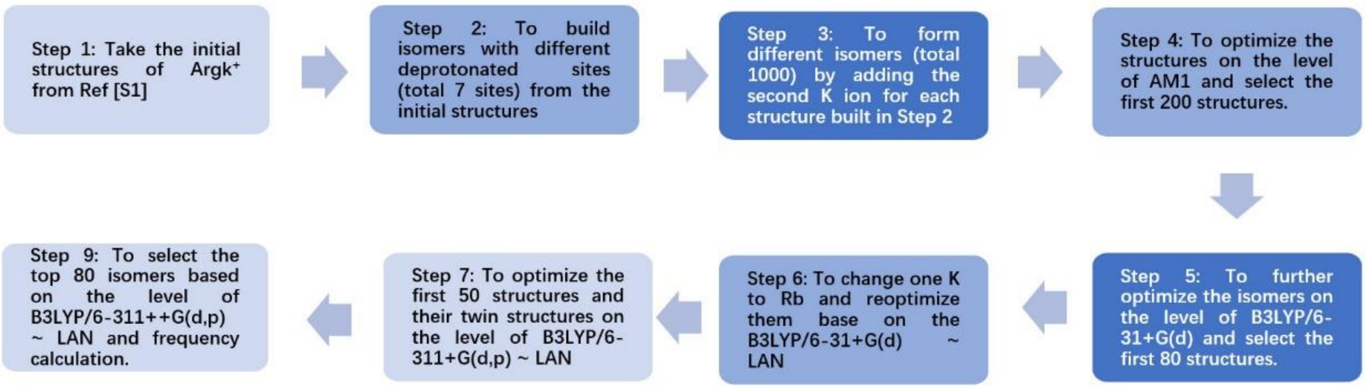

Scheme S1. The self-developed procedure for searching the stable isomers of $[Arg+Rb+K-H]^+$.

Table S1. Relative ZPE-corrected energies (0K) and free energies (298 K) of the 80 isomers in kJ/mol, calculated on the level of B3LYP/6-311++G(d,p)~ LAN.

No	Isomer	ΔE	ΔG	No	Isomer	ΔE	ΔG
1	**1*a***	0	0	41	**22*a***	97.8	96.6
2	**2*a***	5	7.1	42	**20*b***	103.1	96.7
3	**3*a***	6.2	8.2	43	**21*b***	104.7	102.1
4	**4*a***	6.9	8.6	44	**22*b***	113.4	110.3
5	**5*a***	7.1	7.2	45	**23*a***	116.5	122.3
6	**6*a***	8.3	9	46	**23*b***	118.2	122.3
7	**4*b***	8.9	8.2	47	**24*a***	125.5	131.5
8	**5*b***	9.1	9.2	48	**24*b***	125.8	132
9	**7*a***	9.2	11.8	49	**25*a***	126.7	130.1
10	**8*a***	9.5	9.2	50	**26*a***	131	134.3
11	**6*b***	10	9.6	51	**25*b***	135.1	139
12	**1*b***	11	10.1	52	**26*b***	136.6	141.4
13	**9*a***	11.3	16.3	53	**27*a***	142.1	145.8
14	**10*a***	11.8	11.1	54	**27*b***	145.6	149.7
15	**11*a***	12.2	13.6	55	**28*a***	149.6	153.5
16	**9*b***	12.2	17.2	56	**29*a***	151.9	155.1
17	**3*b***	12.3	13.6	57	**28*b***	151.9	155.1
18	**12*a***	12.8	11.6	58	**30*a***	152	151.7
19	**13*a***	13.3	11.6	59	**29*b***	154.1	157.2
20	**8*b***	16.4	18.2	60	**30*b***	155.7	155.7
21	**14*a***	16.8	18.8	61	**31*a***	168	168.9
22	**2*b***	17	23.4	62	**32*a***	169.9	169.7
23	**13*b***	18.1	15.6	63	**31*b***	173.7	173.6
24	**11*b***	18.3	18.8	64	**33*a***	176.8	182.3
25	**15*a***	18.7	18.3	65	**32*b***	178.1	177.6
26	**10*b***	19	19.4	66	**34*a***	181.9	179.7
27	**12*b***	19.4	18.4	67	**33*b***	182.5	188.5
28	**7*b***	19.8	22.8	68	**34*b***	182.7	179.8
29	**14*b***	20.8	23.8	69	**35*a***	187	184.7
30	**16*a***	22.8	22.5	70	**36*a***	189.6	181.6
31	**17*a***	23.6	22.7	71	**37*a***	191.1	185.1
32	**15*b***	25.8	23.6	72	**38*a***	194.4	193.3
33	**16*b***	29	26.9	73	**38*b***	194.4	192.8
34	**17*b***	29.8	29.9	74	**39*a***	195.4	193.6
35	**18*a***	58.5	56.7	75	**39*b***	196.2	193.8
36	**19*a***	73.6	70.2	76	**35*b***	207.5	207.7
37	**18*b***	82.2	80.1	77	**37*b***	208.9	203
38	**19*b***	82.9	82	78	**36*b***	216.6	210.4
39	**20*a***	87.1	79.3	79	**40*a***	217.6	224.7
40	**21*a***	95.5	92.9	80	**40*b***	219.8	227.1

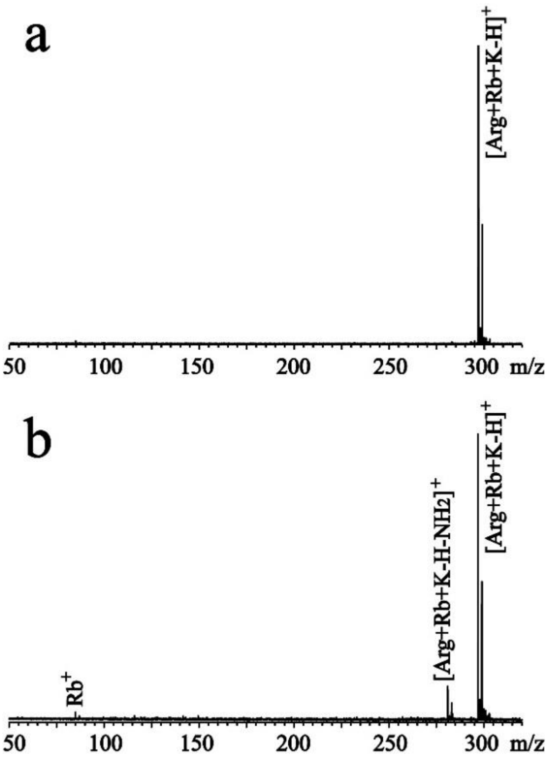

Figure S1. (a) The mass spectrum of $[Arg+Rb+K{-}H]^+$ after the isolation, (**b**) the IRMPD mass spectrum after the irradiation at 3065 cm^{-1} for 40s.

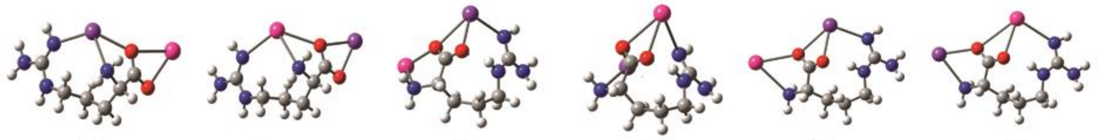

1*a* 1*b* 2*a* 2*b* 3*a* 3*b*

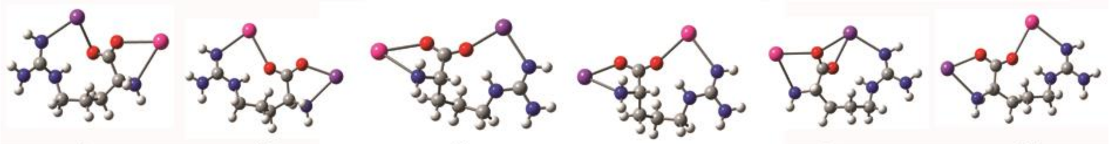

4*a* 4*b* 5*a* 5*b* 6*a* 6*b*

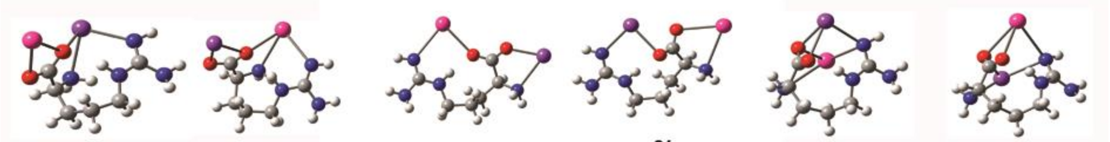

7*a* 7*b* 8*a* 8*b* 9*a* 9*b*

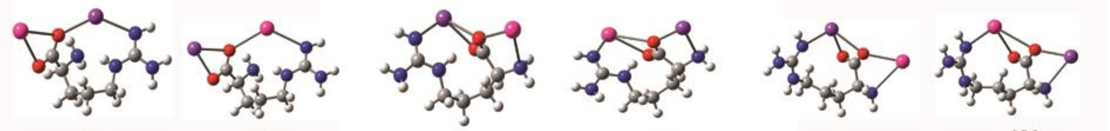

10*a* 10*b* 11*a* 11*b* 12*a* 12*b*

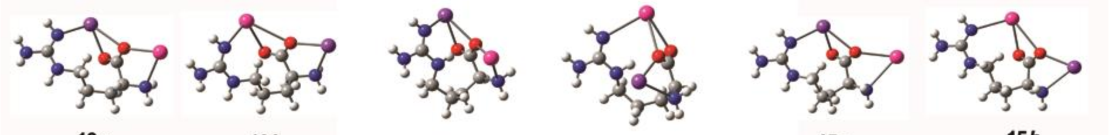

13*a* 13*b* 14*a* 14*b* 15*a* 15*b*

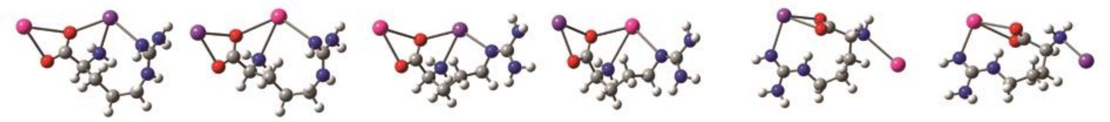

16*a* 16*b* 17*a* 17*b* 18*a* 18*b*

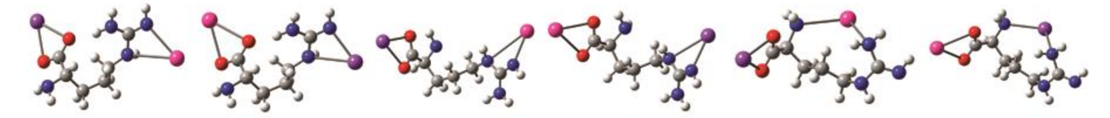

19*a* 19*b* 20*a* 20*b* 21*a* 21*b*

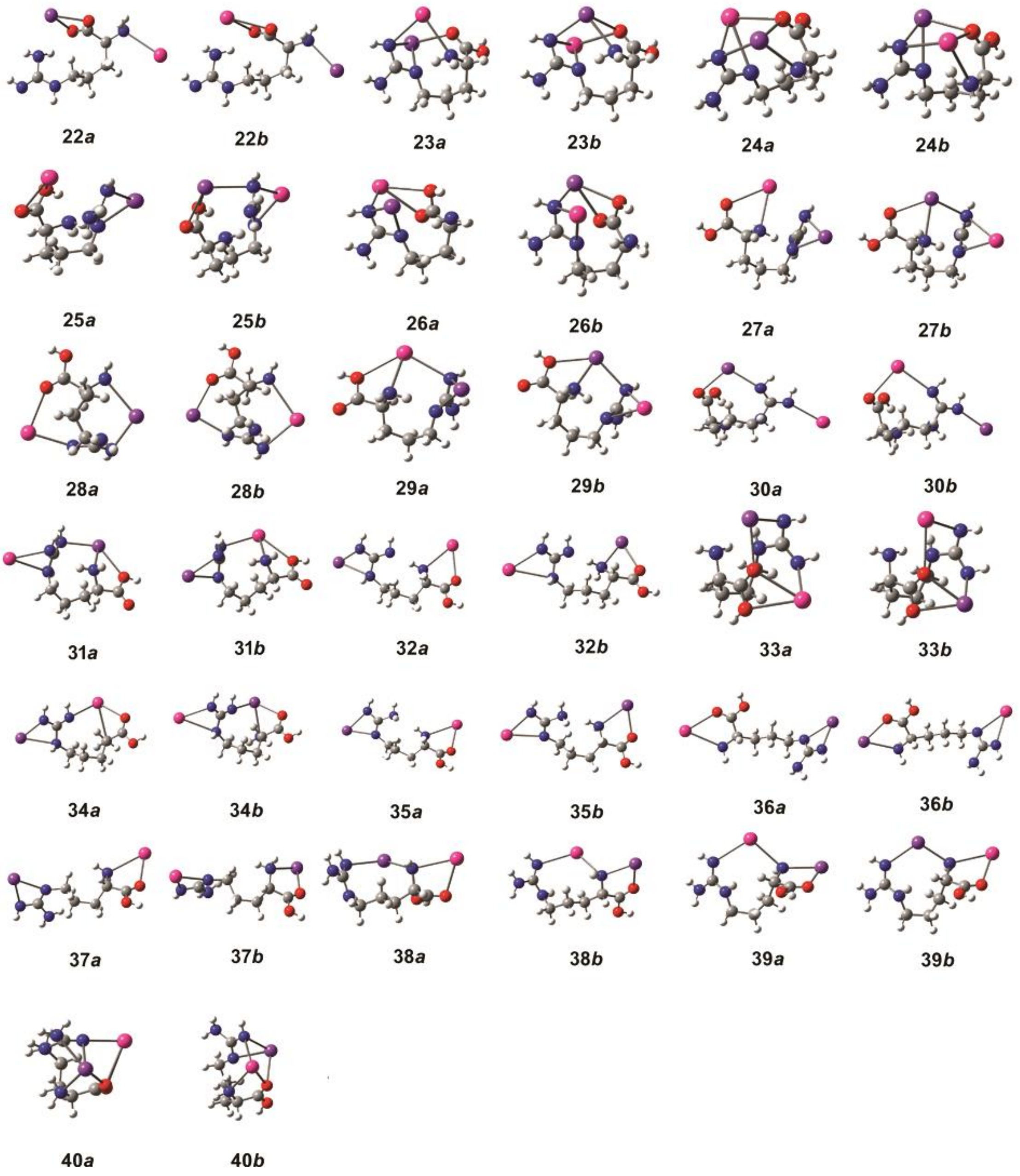

Figure S2 The structures of the top 80 isomers. Their relative energies are shown in Table S1.

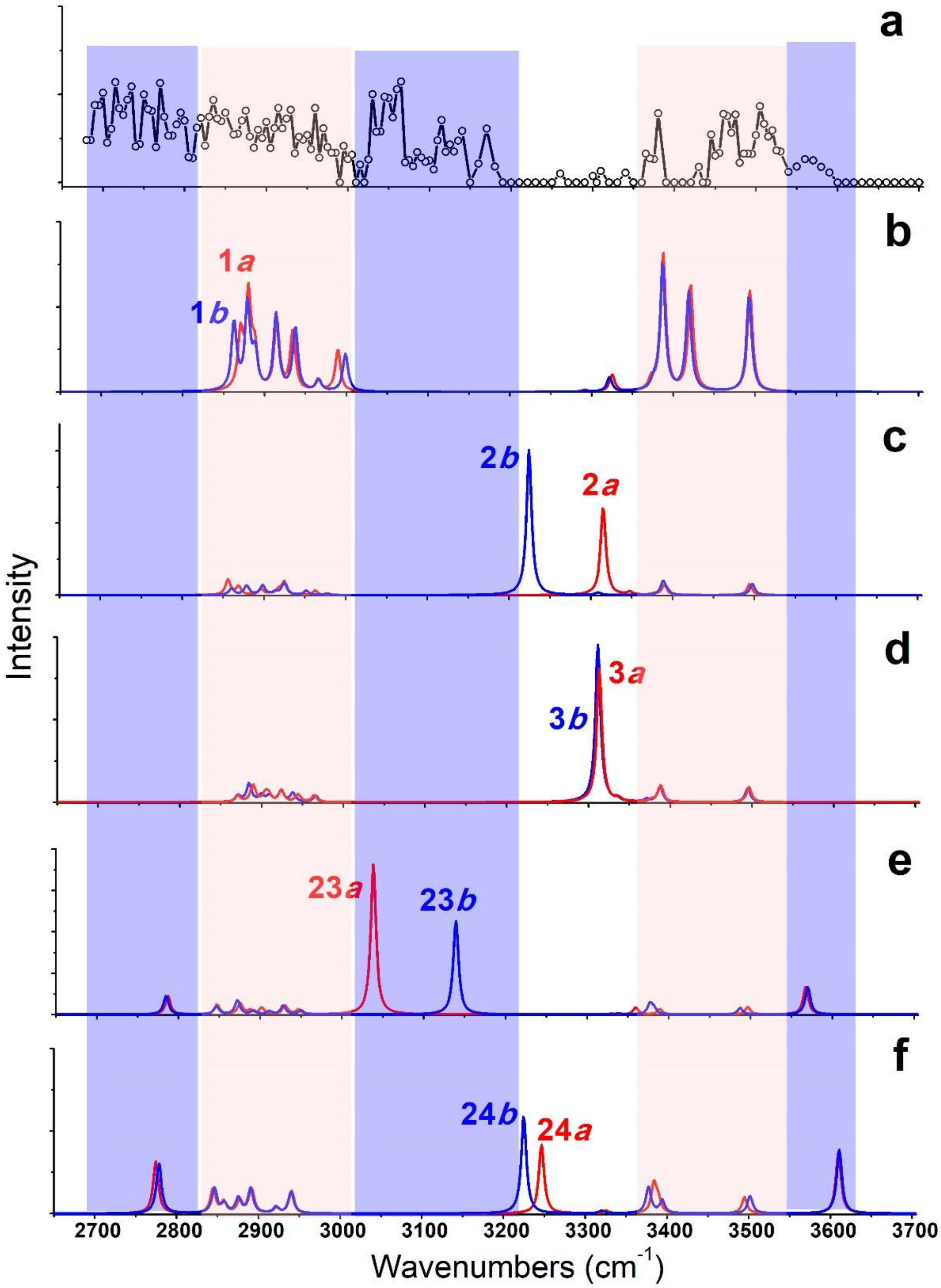

Figure S3. Experimental IRMPD spectrum of [Arg+Rb+K−H]+ (**a**) and the calculated vibrational spectra of different isomers obtained at the level of MP2/6-311++G(d,p)~SDD (**b**–**f**).

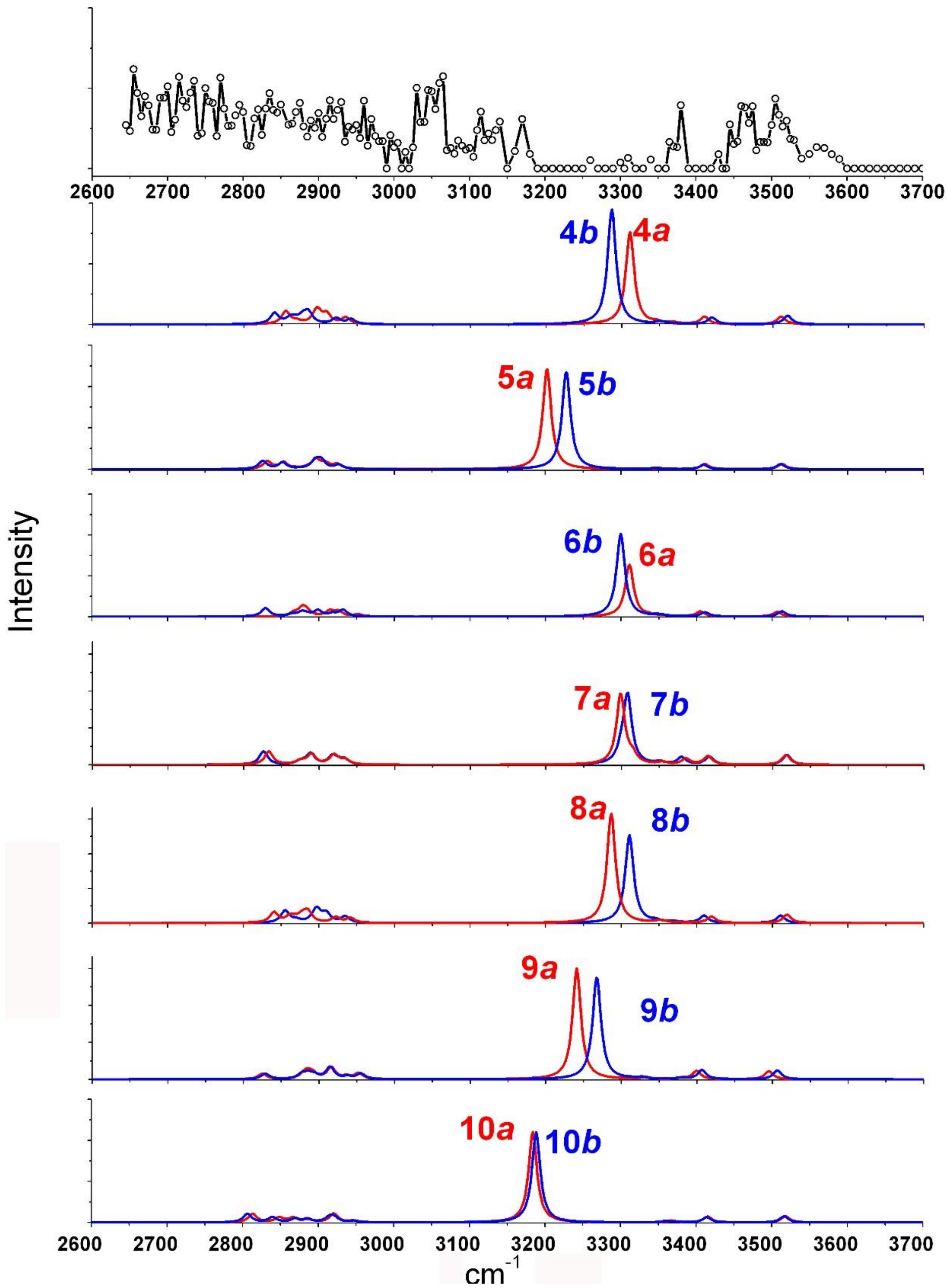

2600
2700
2800
2900
3000
3100
3200
3300
3400
3500
3600
3700
4b
4a
5a
5b
6b
6a
7a
7b
8a
8b
9a
9b
10a
10b
Intensity
cm-1

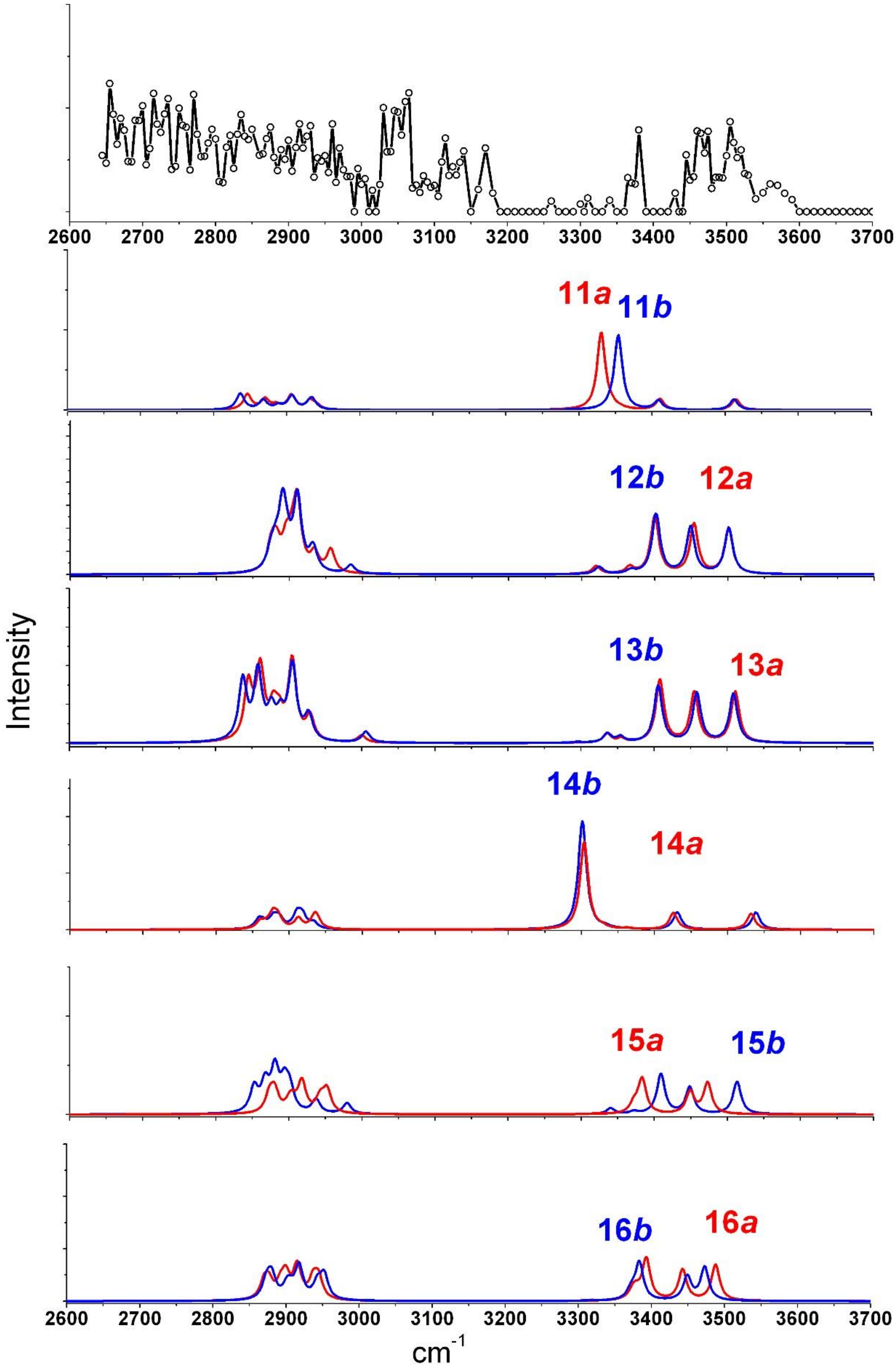

Figure S4. The predicted vibrational spectra of the top isomers of **4*a*/*b*** ~ **16*a*/*b*.**

Table S2. Cartesian coordinates of some structures.

Isomers	No	Atomic number	x	y	z
	1	7	0.394405	1.800752	1.743674
	2	6	-0.356062	2.090510	0.497980
	3	6	-1.362941	0.964892	0.147186
	4	6	0.582649	2.371924	-0.690619
	5	8	-1.338909	-0.123991	0.811933
	6	8	-2.155090	1.223779	-0.781833
	7	6	1.522190	1.235250	-1.139319
	8	6	2.869712	1.192019	-0.398150
	9	7	3.811518	0.216862	-0.933235
	10	6	3.898379	-1.083062	-0.522012
	11	7	4.851485	-1.813879	-1.214096
	12	7	3.130319	-1.564550	0.409746
1*a*	13	1	1.035593	2.569689	1.926613
	14	1	-0.261387	1.806178	2.521975
	15	1	-0.975442	2.988982	0.613644
	16	1	1.175390	3.268205	-0.465234
	17	1	-0.068780	2.640966	-1.523693
	18	1	1.736989	1.372576	-2.204554
	19	1	1.022221	0.263809	-1.062843
	20	1	2.740414	0.980106	0.662527
	21	1	3.348059	2.174363	-0.465493
	22	1	4.212787	0.426117	-1.834704
	23	1	5.038385	-2.744847	-0.875525
	24	1	5.678369	-1.325105	-1.526260
	25	1	3.437325	-2.504747	0.649064
	26	37	-3.924908	-0.904457	-0.508787
	27	19	0.885055	-0.965956	1.829253
	1	7	-0.600141	0.518917	2.184017
	2	6	-1.300534	1.460081	1.282084
	3	6	-2.130484	0.729679	0.200980
	4	6	-0.338666	2.481099	0.646093
	5	8	-2.088970	-0.541416	0.116430
	6	8	-2.839696	1.464212	-0.526345
	7	6	0.786256	1.939952	-0.258581
	8	6	2.079944	1.563480	0.484743
	9	7	3.211834	1.299174	-0.394285
	10	6	3.559799	0.067573	-0.875467
1*b*	11	7	4.655866	0.107812	-1.725993
	12	7	2.896823	-1.007373	-0.572760
	13	1	-0.095974	1.056029	2.885969
	14	1	-1.298536	-0.012278	2.699227
	15	1	-2.043357	2.053635	1.832539
	16	1	0.100476	3.090066	1.447087
	17	1	-0.966850	3.155857	0.062473
	18	1	1.039899	2.721792	-0.982504
	19	1	0.435723	1.089831	-0.853552
	20	1	1.940788	0.690040	1.119386
	21	1	2.367100	2.390134	1.142922
	22	1	3.578977	2.090933	-0.900240

	23	1	5.029029	-0.781922	-2.018352
	24	1	5.368284	0.797535	-1.533551
	25	1	3.392404	-1.819185	-0.934961
	26	19	-4.192078	-0.383736	-1.556933
	27	37	0.355197	-1.959317	0.637260
**2*a***	1	7	-2.343208	1.520379	-1.090508
	2	6	-0.877689	1.477222	-1.316681
	3	6	-0.357946	0.021214	-1.368544
	4	6	-0.172313	2.272982	-0.190656
	5	8	0.714617	-0.153306	-1.999648
	6	8	-0.985658	-0.885464	-0.744901
	7	6	1.292249	2.696685	-0.473063
	8	6	2.347298	2.069290	0.446863
	9	7	2.455231	0.637388	0.203768
	10	6	2.988192	-0.299235	1.032337
	11	7	3.618637	0.192256	2.163408
	12	7	2.835617	-1.567451	0.761848
	13	1	-2.805885	1.076141	-1.881250
	14	1	-2.644957	2.492309	-1.115201
	15	1	-0.608837	1.934445	-2.275243
	16	1	-0.223057	1.694979	0.740008
	17	1	-0.768558	3.175317	-0.017486
	18	1	1.380384	3.780717	-0.366493
	19	1	1.555884	2.471317	-1.510342
	20	1	2.078266	2.245440	1.494536
	21	1	3.306278	2.574594	0.266244
	22	1	2.063678	0.294681	-0.667808
	23	1	4.137587	-0.479748	2.707207
	24	1	4.069767	1.093128	2.099844
	25	1	3.368628	-2.139513	1.410996
	26	19	1.215768	-2.576147	-1.177213
	27	37	-3.057381	-0.482806	1.091548
**2*b***	1	7	-1.914387	-2.649786	0.389265
	2	6	-1.611424	-1.782273	-0.770978
	3	6	-0.107123	-1.400175	-0.750196
	4	6	-2.596908	-0.585601	-0.709299
	5	8	0.316589	-0.682738	-1.690907
	6	8	0.597321	-1.808066	0.219383
	7	6	-2.488587	0.519616	-1.786249
	8	6	-2.043829	1.876592	-1.215424
	9	7	-0.732805	1.752206	-0.568830
	10	6	-0.378883	2.204238	0.659952
	11	7	-1.098867	3.279627	1.135356
	12	7	0.569504	1.589799	1.336794
	13	1	-1.290650	-3.452291	0.382484
	14	1	-2.861509	-3.011661	0.312239
	15	1	-1.768396	-2.295499	-1.729170
	16	1	-2.520644	-0.130885	0.286814
	17	1	-3.601048	-1.020045	-0.744793
	18	1	-3.460026	0.671747	-2.263757
	19	1	-1.793700	0.217106	-2.571243
	20	1	-2.771893	2.224681	-0.477416
	21	1	-2.007214	2.618131	-2.020840

	22	1	-0.166510	0.992636	-0.939462
	23	1	-0.784747	3.704721	1.993399
	24	1	-1.496786	3.924790	0.468986
	25	1	0.850342	2.161160	2.131694
	26	37	2.826579	0.003837	-0.442562
	27	19	-0.559237	-1.032496	2.352796
	1	7	-1.822554	-1.821565	-0.705790
	2	6	-0.668289	-1.572255	0.188822
	3	6	-0.509356	-0.056338	0.465906
	4	6	0.657623	-2.175060	-0.357262
	5	8	0.355494	0.273218	1.314368
	6	8	-1.218281	0.765271	-0.191078
	7	6	1.767611	-2.456143	0.687821
	8	6	3.127859	-1.837663	0.340135
	9	7	3.042256	-0.382032	0.414231
	10	6	3.735074	0.517013	-0.334094
	11	7	4.860116	0.023736	-0.968791
	12	7	3.297605	1.743027	-0.447493
	13	1	-1.928255	-2.826271	-0.827762
**3*a***	14	1	-1.594371	-1.454401	-1.628396
	15	1	-0.895370	-2.049766	1.147685
	16	1	1.039186	-1.512871	-1.143259
	17	1	0.399315	-3.113928	-0.856985
	18	1	1.908745	-3.535016	0.794120
	19	1	1.464211	-2.085603	1.670245
	20	1	3.431739	-2.139315	-0.667969
	21	1	3.881241	-2.221796	1.039932
	22	1	2.219314	-0.021133	0.886835
	23	1	5.453111	0.700678	-1.422646
	24	1	5.363274	-0.730586	-0.525634
	25	1	3.979831	2.323459	-0.927866
	26	37	-3.993736	0.246327	-0.189042
	27	19	0.848890	2.635991	0.278855
	1	7	-2.728096	-1.907076	-0.666135
	2	6	-1.546228	-1.731119	0.216383
	3	6	-1.233493	-0.224305	0.432206
	4	6	-0.294570	-2.496180	-0.300007
	5	8	-0.277272	0.044179	1.202554
	6	8	-1.934039	0.644691	-0.186056
	7	6	0.777135	-2.847128	0.765237
	8	6	2.205160	-2.445070	0.367977
	9	7	2.315765	-0.991020	0.334009
**3*b***	10	6	3.224023	-0.257083	-0.367909
	11	7	4.289282	-0.974868	-0.891146
	12	7	3.030200	1.025012	-0.547018
	13	1	-2.967147	-2.897646	-0.692823
	14	1	-2.451931	-1.670020	-1.620084
	15	1	-1.821332	-2.139768	1.197194
	16	1	0.158914	-1.909260	-1.110204
	17	1	-0.650761	-3.424297	-0.763616
	18	1	0.773039	-3.926146	0.953285
	19	1	0.537374	-2.361888	1.716581
	20	1	2.452756	-2.862009	-0.617354

	21	1	2.908614	-2.878243	1.095101
	22	1	1.534275	-0.478926	0.735290
	23	1	5.037904	-0.432060	-1.296038
	24	1	4.605841	-1.792615	-0.388382
	25	1	3.839564	1.446400	-0.998831
	26	19	-4.453239	0.258170	-0.285433
	27	37	0.573046	2.563548	0.129455
**23*a***	1	6	1.512053	-1.629699	-0.870625
	2	6	2.801706	-0.780266	-1.088429
	3	6	2.924514	0.606572	-0.424587
	4	6	1.906116	1.695910	-0.805919
	5	7	0.611699	1.412161	-0.206596
	6	6	-0.404592	2.265392	-0.335643
	7	7	-0.244813	3.475200	-1.002570
	8	7	-1.573929	1.960403	0.261427
	9	1	0.538621	3.585571	-1.626555
	10	1	-1.072089	3.982749	-1.273573
	11	1	-2.233641	2.728351	0.164691
	12	1	2.031344	-2.686051	2.119176
	13	1	1.732921	-2.623770	-1.275715
	14	1	3.655252	-1.383190	-0.765685
	15	1	2.902002	-0.668019	-2.173942
	16	1	2.915778	0.496313	0.667854
	17	1	3.925144	0.975004	-0.674093
	18	1	2.317477	2.659908	-0.465543
	19	1	1.861392	1.759644	-1.909335
	20	7	0.308427	-1.123903	-1.532794
	21	1	0.461975	-1.158978	-2.536726
	22	6	1.235559	-1.872412	0.606008
	23	8	2.257233	-2.511732	1.192543
	24	8	0.234785	-1.556682	1.228093
	25	37	-2.456588	-0.854306	-0.383984
	26	19	-0.092689	1.031452	2.322545
	27	1	0.235507	-0.132131	-1.253073
**23*b***	1	6	1.887195	-1.191088	-1.014419
	2	6	2.911959	-0.023216	-1.128692
	3	6	2.652276	1.288511	-0.361164
	4	6	1.389749	2.102399	-0.697580
	5	7	0.198860	1.447476	-0.175938
	6	6	-0.983796	2.059622	-0.238897
	7	7	-1.102921	3.334177	-0.793612
	8	7	-2.051931	1.456398	0.316154
	9	1	-0.392172	3.639774	-1.440433
	10	1	-2.030490	3.653708	-1.026985
	11	1	-2.883729	2.032439	0.216402
	12	1	2.608228	-2.291193	1.916354
	13	1	2.367136	-2.064990	-1.468667
	14	1	3.888962	-0.404972	-0.819321
	15	1	2.995308	0.196613	-2.198868
	16	1	2.655159	1.089549	0.718938
	17	1	3.524037	1.925621	-0.544611
	18	1	1.529501	3.107842	-0.267578
	19	1	1.356712	2.251192	-1.793927

	20	7	0.611679	-0.971317	-1.699612
	21	1	0.764512	-1.023965	-2.701936
	22	6	1.653644	-1.600522	0.434402
	23	8	2.801161	-1.975952	1.020217
	24	8	0.593670	-1.616531	1.035507
	25	19	-1.793054	-1.147089	-0.338461
	26	37	-0.322450	1.047091	2.737610
	27	1	0.316313	-0.015843	-1.458031
	1	6	-0.786392	2.332829	-0.808946
	2	6	-0.977378	2.942801	0.615313
	3	6	-0.493972	2.168376	1.861086
	4	6	-1.011591	0.736281	2.065186
	5	7	-0.483305	-0.140790	1.032829
	6	6	-0.708349	-1.453345	1.100888
	7	7	-1.377381	-2.008009	2.192802
	8	7	-0.221275	-2.252634	0.133142
	9	1	-1.959391	-1.408912	2.756912
	10	1	-1.731250	-2.946833	2.093843
	11	1	-0.420063	-3.227151	0.346114
	12	1	1.291725	3.384435	-0.113325
**24*a***	13	1	-0.937722	3.153398	-1.524026
	14	1	-0.520629	3.943057	0.640771
	15	1	-2.050751	3.137780	0.707898
	16	1	0.601764	2.129390	1.876108
	17	1	-0.781659	2.769601	2.729994
	18	1	-0.705743	0.429203	3.078838
	19	1	-2.118225	0.762618	2.082386
	20	7	-1.734967	1.274044	-1.156403
	21	1	-2.667570	1.668664	-1.228756
	22	6	0.641720	1.854440	-1.085496
	23	8	1.637926	2.624013	-0.599925
	24	8	0.949273	0.869614	-1.720070
	25	19	-0.762053	-1.129223	-2.266434
	26	37	2.419440	-0.820474	0.397824
	27	1	-1.706723	0.611829	-0.372924
	1	6	-2.499812	0.149118	-0.513439
	2	6	-2.956021	-0.315624	0.905597
	3	6	-1.907251	-0.559374	2.012595
	4	6	-0.792443	-1.580782	1.736135
	5	7	0.125692	-1.063245	0.734767
	6	6	1.212991	-1.758752	0.394986
	7	7	1.528829	-2.951037	1.039188
	8	7	2.039132	-1.249510	-0.538527
**24*b***	9	1	0.812508	-3.431235	1.559871
	10	1	2.214762	-3.554354	0.614017
	11	1	2.870930	-1.828361	-0.627061
	12	1	-2.746308	2.151621	0.839287
	13	1	-3.403467	0.497962	-1.032436
	14	1	-3.697552	0.396461	1.295708
	15	1	-3.527922	-1.235458	0.743128
	16	1	-1.431805	0.388489	2.292480
	17	1	-2.463495	-0.885668	2.897600
	18	1	-0.294826	-1.782038	2.698693

19	1	-1.261439	-2.536456	1.434125
20	7	-1.893100	-0.895424	-1.337913
21	1	-2.578225	-1.629381	-1.491749
22	6	-1.579224	1.371516	-0.477961
23	8	-1.937767	2.366642	0.354447
24	8	-0.573006	1.517196	-1.138879
25	37	0.580972	-0.458385	-3.073886
26	19	1.616048	1.182879	0.566533
27	1	-1.123582	-1.275359	-0.768837

Reference

[33] Bush, M. F.; O'Brien, J. T.; Prell, J. S.; Saykally, R. J.; Williams, E. R. Infrared spectroscopy of cationized arginine in the gas phase: Direct evidence for the transition from nonzwitterionic to zwitterionic structure. *J. Am. Chem. Soc.* **2007**, *129*, 1612–1622.
